# Pan-cancer analysis of prognostic and immunological role of DTYMK in human tumors

**DOI:** 10.3389/fgene.2022.989460

**Published:** 2022-09-08

**Authors:** Huihui Zhao, Rongrong Xie, Chenxi Zhang, Guojun Lu, Hui Kong

**Affiliations:** ^1^ Department of Oncology, The Second Hospital of Nanjing, Nanjing University of Chinese Medicine, Nanjing, China; ^2^ Department of Respiratory and Critical Care Medicine, The First Affiliated Hospital of Nanjing Medical University, Nanjing, China; ^3^ Central Laboratory, Nanjing Chest Hospital, Affiliated Nanjing Brain Hospital, Nanjing Medical University, Nanjing, China; ^4^ Department of Respiratory Medicine, Nanjing Chest Hospital, Affiliated Nanjing Brain Hospital, Nanjing Medical University, Nanjing, China

**Keywords:** DTYMK, immunotherapy, TCGA, prognosis, immune infiltrates

## Abstract

**Background:** Deoxythymidylate kinase (DTYMK) has been reported to correlate with the progression of hepatocellular carcinoma. However, the role of DTYMK in human cancers is not studied. In this study, we studied the prognostic value, functional states, and correlations with immune infiltration of DTYMK in human cancers.

**Methods:** The Cancer Genome Atlas (TCGA), Genotype-Tissue Expression (GTEx), UALCAN, Clinical Proteomic Tumor Analysis Consortium (CPTAC), the search tool for the retrieval of interacting genes (STRING), GeneMANIA, cBioPortal, Cancer Single-cell State Atlas (CancerSEA), and Tumor IMmune Estimation Resource (TIMER) databases were utilized to analyze DTYMK in cancers.

**Results:** In general, DTYMK is abnormally expressed between most human cancer and normal tissues from a pan-cancer perspective. DTYMK can be used as a diagnostic biomarker to differentiate tumor tissues from normal tissues in most tumors. Upregulation of DTYMK predicted poor survival status in most cancer types in TCGA. Moreover, DTYMK expression was correlated with the T stage in ACC, BRCA, KIRC, LIHC, and LUAD, with the N stage in BLCA, HNSC, KICH, KIRC, LUAD, LUSC, and THCA, with the M stage in ACC, KIRC, KIRP, and LUAD, with TNM stage in ACC, KIRC, LIHC, LUAD, and LUSC. In addition, based on single-cell sequencing data, we concluded that the expression of DTYMK was correlated with the functional status of the cell cycle, DNA damage, DNA repair, invasion, EMT, and proliferation. Finally, DTYMK expression was correlated with six infiltrating immune cells, including B cells, CD4^+^ T cells, CD8^+^ T cells, neutrophils, macrophages, and dendritic cells by investigating TIMER.

**Conclusion:** Our findings suggested that abnormally expressed DTYMK was correlated with poor survival, malignant functional status, and immune infiltrates. DTYMK might be served as a potential biomarker for diagnosis and poor prognosis in various cancer types. DTYMK might act as a potential target for immune therapy.

## 1 Introduction

The global incidence of cancer is substantial and growing, and it is estimated that every year there are about 23.6 million new cancer cases and 10.0 million cancer deaths globally (Kocarnik et al., 2022). As a result, cancer is becoming an enormous disease burden globally for public health systems (Hwangbo et al., 2018). A previous study has reported that female breast cancer is the most commonly diagnosed cancer, followed by lung cancer and colorectal cancer. Lung cancer is the leading cause of cancer-related death, followed by colorectal cancer and liver cancer (Sung et al., 2021). Accordingly, given that the incidence and burden of cancer are rising globally, it is crucial to identify biomarkers for early diagnosis and prognosis prediction in various cancers.

Deoxythymidylate kinase (DTYMK) is a nuclear-encoded deoxythymidylate kinase and catalyzes the conversion of dTMP to dTDP. The expression of DTYMK can be detected in all tissues and is the key enzyme to catalyze the last reaction of the dTTP production (Caspi et al., 2016). Overexpression of DTYMK can promote tumor cells proliferation and division. For example, a paper from Zhou et al. (2021) reported that DTYMK can regulate the cell cycle to promote hepatocellular carcinoma proliferation. In addition, it is reported that upregulation of DTYMK is correlated with worse overall survival and disease-free survival (Wang et al., 2020; Zhou et al., 2021). In lung cancer, Liu et al. (2013) reported that depletion of DTYMK can lead to growth inhibition and metabolic disorder in LKB1 mutant related lung cancer. Despite these discoveries, however, these studies only focus the evaluation of DTYMK on a few cancer types, and litter is known regarding the prognostic and immunological role of DTYMK in various cancers.

In the present study, we determined the expression of DTYMK and its correlation with the clinicopathological characteristics and prognosis of patients from a pan-cancer perspective. We further evaluated the association between DTYMK and genetic alteration, functional states at a single-cell level, immune infiltrates. Our findings present novel insights into the functional status of DTYMK from a pan-cancer perspective, linking DTYMK expression with tumor prognosis and providing a potential therapeutic target for various cancers.

## 2 Materials and methods

### 2.1 Data collection

The RNA sequence data and corresponding clinical data of The Cancer Genome Atlas (TCGA, https://portal.gdc.cancer.gov/) database and Genotype-Tissue Expression (GTEx) were downloaded from the UCSC Xena database (https://xenabrowser.net/datapages). The workflow type of mRNA data format was converted from Fragments Per Kilobase per Million (FPKM) into transcripts per million reads (TPM) for further analysis. DTYMK expression data in 22 tumor cell lines were downloaded from the Cancer Cell Line Encyclopedia (CCLE, (https://portals.broadinstitute.org/ccle/).

### 2.2 Promoter methylation and protein expression

UALCAN (http://ualcan.path.uab.edu/) is a user-friendly web resource for analyzing cancer OMICS data (Chandrashekar et al., 2017). In this study, we conducted UALCAN to explore the promoter methylation level of DTYMK with TCGA samples and protein expression with the Clinical Proteomic Tumor Analysis Consortium (CPTAC) samples (Edwards et al., 2015).

### 2.3 Prediction of protein-protein interactions and genetic alteration

The search tool for the retrieval of interacting genes (STRING) database (http://string-db.org) is a precomputed online resource and can be used to explore and analyze all publicly available sources of PPI information (Szklarczyk et al., 2019). GeneMANIA (http://www.genemania.org) is a flexible website using available genomics and proteomics data to generate predictions about gene function (Franz et al., 2018). cBioPortal web (https://www.cbioportal.org/) can be used to study genetic alteration characteristics (Cerami et al., 2012; Gao et al., 2013). In this study, we conducted STRING, GeneMANIA, and cBioPortal to predict a PPI network and study the genetic alteration of DTYMK.

### 2.4 Diagnostic and prognostic value analysis

ROC curve was utilized to assess the diagnostic value of DTYMK to distinguish tumors from normal tissues in pan-cancer. Kaplan-Meier analysis was employed to capture the prognostic significance of DTYMK for overall survival (OS), disease-specific survival (DSS), and progress-free interval (PFI).

### 2.5 Cancer single-cell state atlas

CancerSEA (http://biocc.hrbmu.edu.cn/CancerSEA/home.jsp) is the first dedicated resource to comprehensively decode the distinct functional states of cancer cells at the single-cell level (Yuan et al., 2019). CancerSEA can provide a cancer single-cell functional state atlas, including 14 functional states (stemness, invasion, metastasis, proliferation, EMT, angiogenesis, apoptosis, cell cycle, differentiation, DNA damage, DNA repair, hypoxia, inflammation, and quiescence) from 25 cancer types. In the present study, CancerSEA was used to explore the expression profile of DTYMK at a single-cell level and its potential functional status in pan-cancer.

### 2.6 Immune infiltrates analysis

Tumor Immune Estimation Resource (TIMER) is an online server for a comprehensive overview of immune infiltrates across multiple cancer types (Li et al., 2016; Li et al., 2017). The abundances of six infiltrating immune cells, including B cells, CD4^+^ T cells, CD8^+^ T cells, neutrophils, macrophages, and dendritic cells, are estimated by the TIMER algorithm. In this study, we first used the gene module to explore the correlation between DTYMK expression and the abundance of six infiltrating immune cells. Then we used the survival module to determine the association between clinical outcome and the abundance of six infiltrating immune cells or DTYMK expression. Kaplan-Meier curve parameter was set as split percentage of patients up to 50% percentile. Finally, we used the correlation module to explore correlations between DTYMK expression and gene markers of immune infiltrates.

### 2.7 Statistical analyses

Statistical analyses were performed using R (V 3.6.3) and visualized with R package ggplot2. Paired t-test and Mann-Whitney U test were used to explore DTYMK expression in paired and non-paired samples respectively. The survminer and pROC package (Robin et al., 2011) were used to elucidate the prognostic and diagnostic performance of DTYMK expression. R package survival was performed for multivariate Cox regression analyses.

## 3 Results

### 3.1 Deoxythymidylate kinase mRNA expression in pan-cancer perspective

**FIGURE 1:**
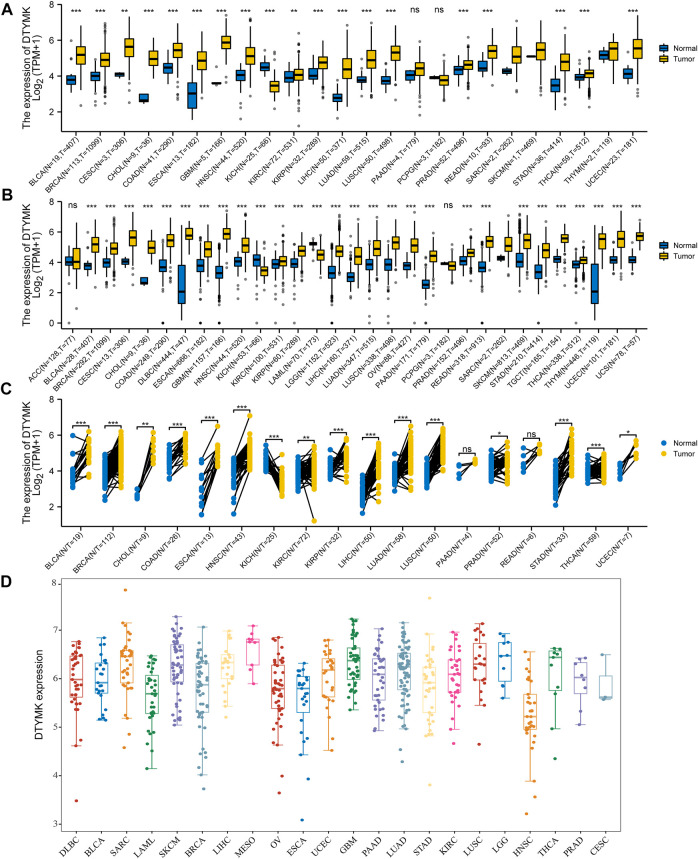
DTYMK expression in Pan-Cancer perspective. **(A)** Differential analysis of DTYMK expression with data from TCGA. **(B)** TCGA and GTEx data indicated differential expression of DTYMK in the Pan-Cancer perspective. **(C)** Paired t-test with TCGA data. **(D)** DTYMK expression in different tumor cell lines from CCLE was in higher ranges. (ns, no significant; *, *p* < 0.05, **, *p* < 0.01, ***, *p* < 0.001).

To elucidate DTYMK mRNA expression in human cancers, we first downloaded the pan-cancer RNA-seq data of DTYMK mRNA expression from TCGA and GTEx, and then eliminated these columns which contained only tumor samples. Abbreviations of tumor names were listed in Supplementary Table S1. As shown in Figure 1A, with only the TCGA database, Mann-Whitney U-test showed the mRNA expression of DTYMK was significantly upregulated in 18 cancer types, while only downregulated in KICH. Furthermore, given the lack of normal samples for some cancers in TCGA, we then integrated the GTEx database. The results in Figure 1B indicated that DTYMK expression was significantly upregulated in 26 cancer types, while only downregulated in KICH and LAML. We further validated DTYMK expression across cancer types with tumor tissues and paired normal tissues from the TCGA database. Paired t-test analysis suggested that DTYMK expression was significantly upregulated in 15 tumor tissues and only downregulated in KICH (Figure 1C). As shown in Figure 1D, DTYMK expression in different tumor cell lines from CCLE was in higher ranges than that of normal tissues in Figure 1B. Taken together, these results suggested that DTYMK was abnormally expressed in pan-cancer perspective.

### 3.2 Protein expression and promoter methylation level of deoxythymidylate kinase

**FIGURE 2:**
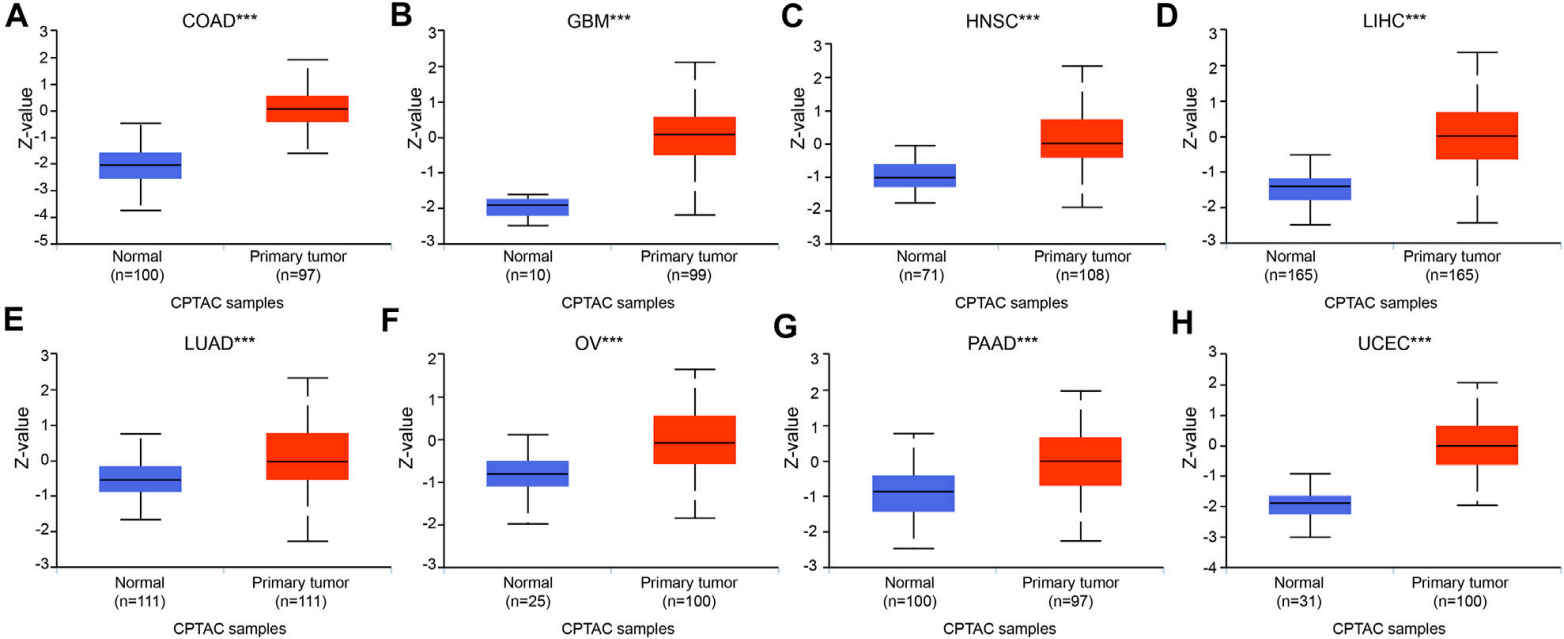
Protein expression of DTYMK. The upregulated protein of DTYMK was detected in COAD **(A)**, GBM **(B)**, HNSC **(C)**, LIHC **(D)**, LUAD **(E)**, OV **(F)**, PAAD **(G)**, and UCEC **(H)**. (***, *p* < 0.001).

**FIGURE 3:**
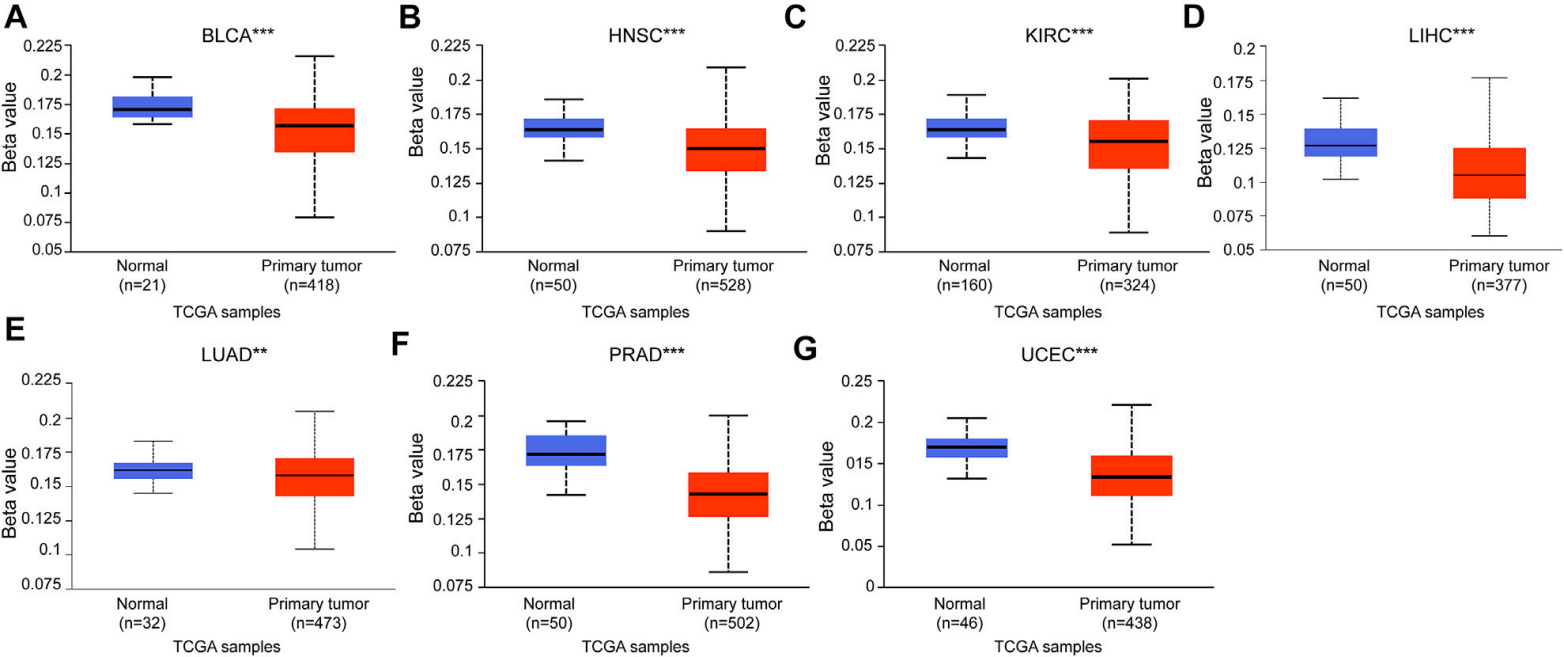
Promoter methylation level of DTYMK. Promoter methylation level of DTYMK was downregulated in BLCA **(A)**, HNSC **(B)**, KIRC **(C)**, LIHC **(D)**, LUAD **(E)**, PRAD **(F)**, and UCEC **(G)**. (**, *p* < 0.01, ***, *p* < 0.001).

To increase the reliability of the DTYMK expression level, we carried out UALCAN to analyze the protein expression of DTYMK between tumor and normal tissues in CPTAC. As shown in Figure 2, in accordance with mRNA expression level, the upregulated protein of DTYMK was detected in COAD, GBM, HNSC, LIHC, LUAD, OV, PAAD, and UCEC. However, the downregulated protein of DTYMK was detected in BRCA and KIRC, while no significant difference was found between different age groups in PRAD (Supplementary Figure S1). The RNA and protein expression of BRCA and KIRC was inconsistency. We speculated the reason for this was that protein was not only regulated at transcription level. Furthermore, in an attempt to compare the promoter methylation level of DTYMK between tumor and normal tissues, we performed UALCAN analysis with TCGA samples. From the data in Figure 3, it was apparent that the promoter methylation level of DTYMK was downregulated in BLCA, HNSC, KIRC, LIHC, LUAD, PRAD, and UCEC. However, no significant difference in promoter methylation level was found in other tumors (Supplementary Figure S2).

### 3.3 Diagnostic value of deoxythymidylate kinase to distinguish tumor from normal tissues

**FIGURE 4:**
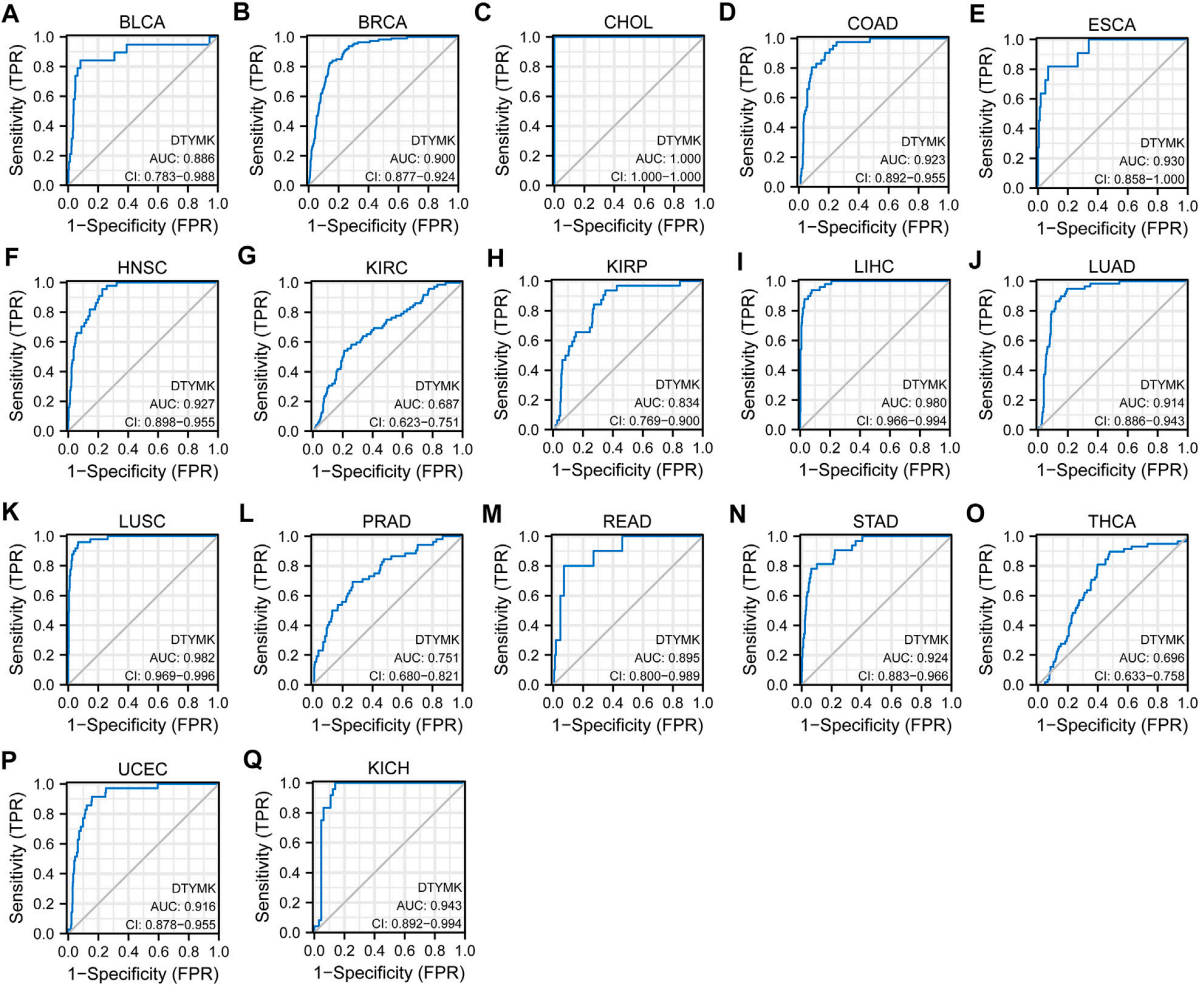
Diagnostic value of DTYMK to distinguish tumor tissues from normal tissues. The AUC value was more than 0.8 in **(A)** BLCA, **(B)** BRCA, **(C)** CHOL, **(D)** COAD, **(E)** ESCA, **(F)** HNSC, **(H)** KIRP, **(I)** LIHC, **(J)** LUAD, **(K)** LUSC, **(M)** READ, **(N)** STAD, **(P)** UCEC, **(Q)** KICH, and less than 0.8 in **(G)** KIRC, **(L)** PRAD, **(O)** THCA.

Based on DTYMK being abnormally expressed from a pan-cancer perspective, we speculated that DTYMK can be used as a diagnostic marker. To validate this hypothesis, we conducted a ROC curve analysis with R package pROC. The results listed in Figure 4 suggested that the AUC value was more than 0.80 in most tumors. The cut-off value, sensitivity, specificity, positive predictive value, negative predictive value, and Youden index of DTYMK were shown in Supplementary Table S2. These results suggested that DTYMK can be used as a diagnostic biomarker to differentiate tumor tissues from normal tissues in most tumors.

### 3.4 Prognostic value of deoxythymidylate kinase in pan-cancer perspective

**FIGURE 5:**
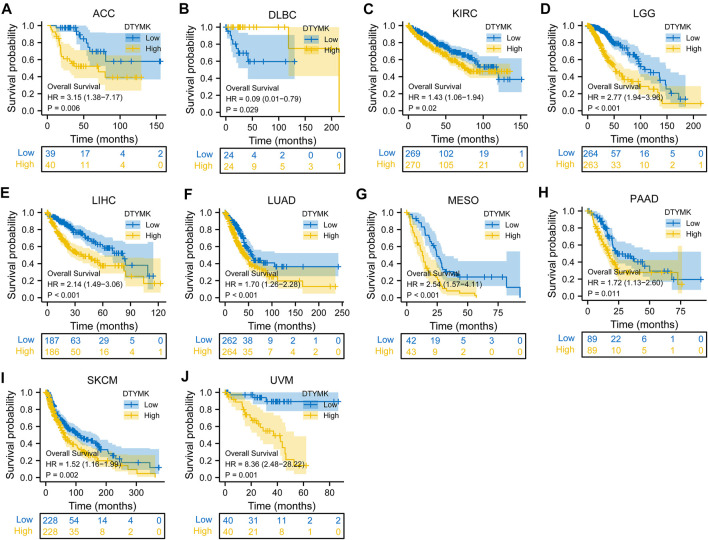
Relationship between DTYMK expression and OS. High DTYMK expression was correlated with short OS in ACC **(A)**, KIRC **(C)**, LGG **(D)**, LIHC **(E)**, LUAD **(F)**, MESO **(G)**, PAAD **(H)**, SKCM **(I)**, and UVM **(J)**. And patients with high DTYMK expression in DLBC had better overall survival **(B)**.

**FIGURE 6:**
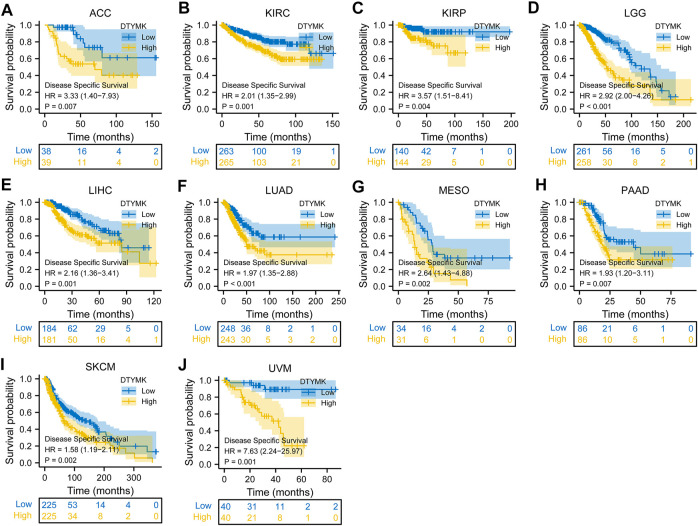
Relationship between DTYMK expression and DSS. High DTYMK expression was correlated with short DSS in ACC **(A)**, KIRC **(B)**, KIRP **(C)**, LGG **(D)**, LIHC **(E)**, LUAD **(F)**, MESO **(G)**, PAAD **(H)**, SKCM **(I)** and UVM **(J)**.

**FIGURE 7:**
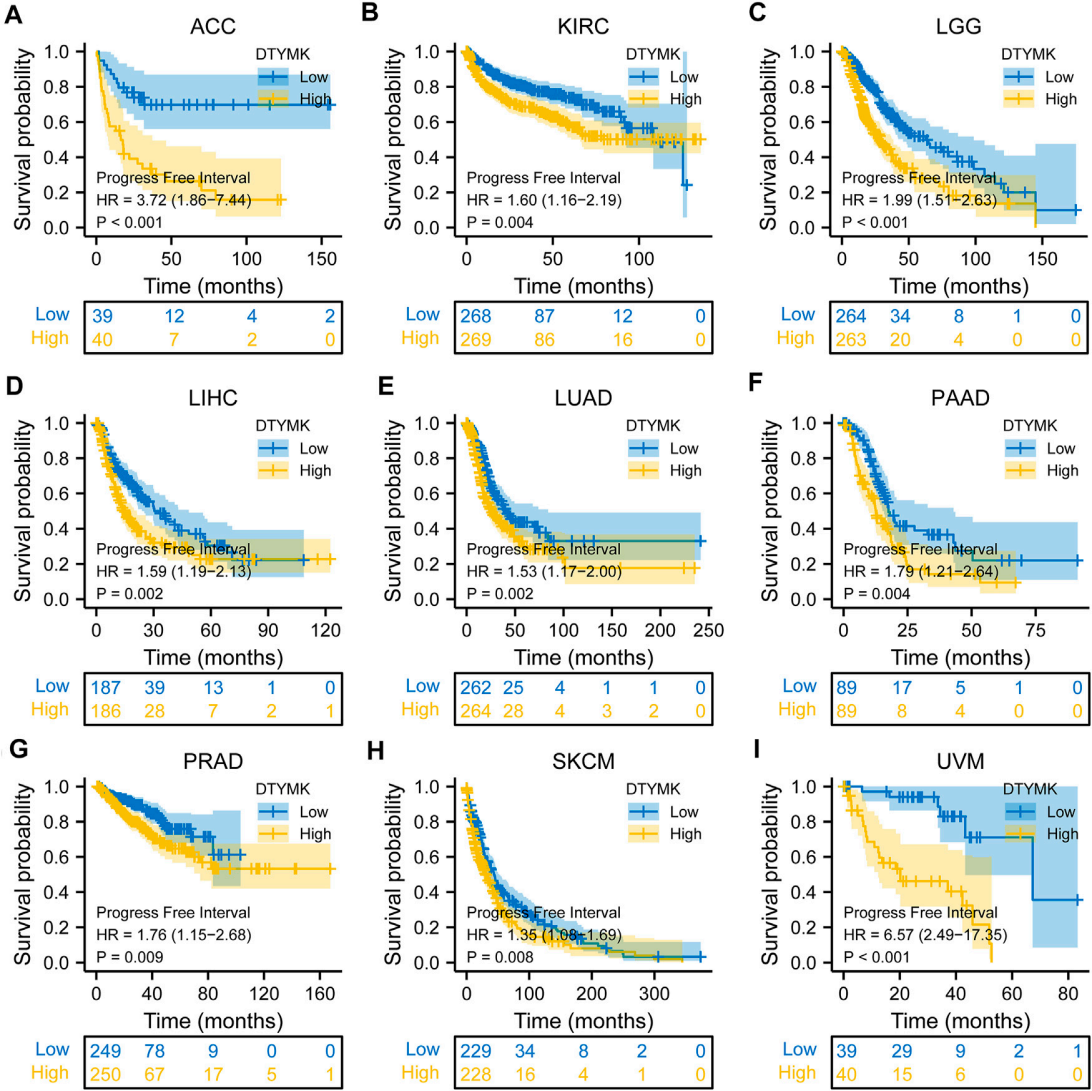
Relationship between DTYMK expression and PFI. DTYMK acted as a risk factor for patients with ACC **(A)**, KIRC **(B)**, LGG **(C)**, LIHC **(D)**, LUAD **(E)**, PAAD **(F)**, PRAD **(G)**, SKCM **(H)**, and UVM **(I)**.

**FIGURE 8:**
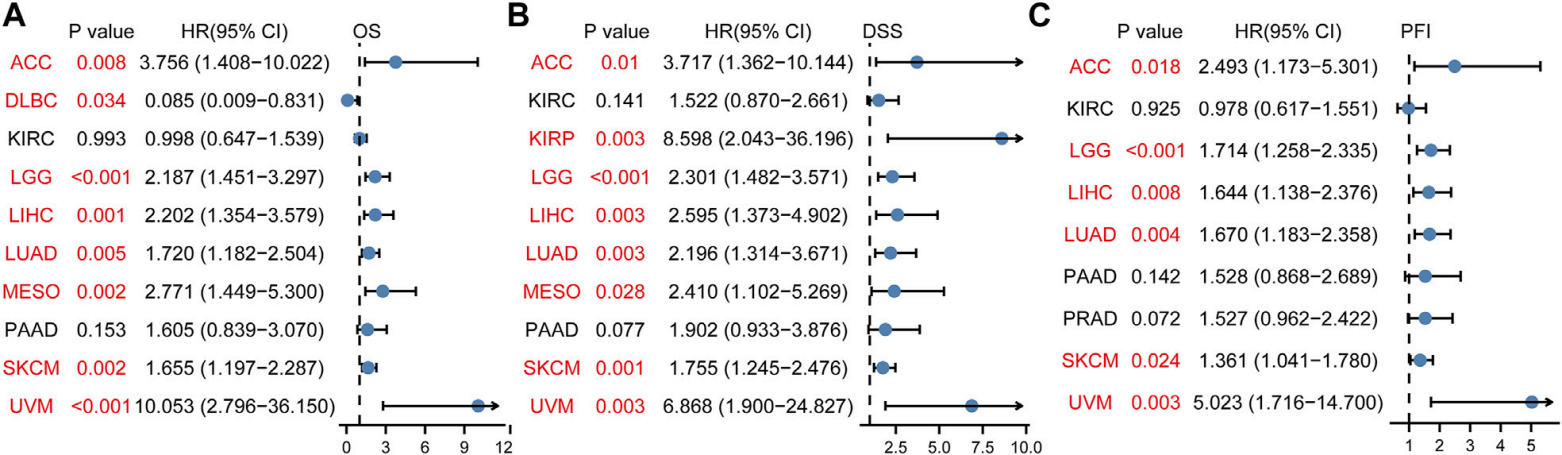
Cox regression analyses of DTYMK in TCGA pan-cancer. **(A)** Forest map indicated the Cox regression results of DTYMK for OS. **(B)** Forest map indicated the Cox regression results of DTYMK for DSS. **(C)** Forest map indicated the Cox regression results of DTYMK for PFI. Red colors mean significant results.

To study the relationship between DTYMK expression and OS, DSS, and PFI, patients with cancer were divided into high/low expression groups according to the median level of DTYMK expression, R package survminer and survival were performed. As shown in Figure 5, patients with high DTYMK expression had short OS than those of patients with low DTYMK expression in ACC (*p* = 0.006), KIRC (*p* = 0.02), LGG (*p* < 0.001), LIHC (*p* < 0.001), LUAD (*p* < 0.001), MESO (*p* < 0.001), PAAD (*p* = 0.011), SKCM (*p* = 0.002), and UVM (*p* = 0.001). And patients with high DTYMK expression in DLBC had better OS (*p* = 0.029). It was apparent from Figure 6 that patients with high DTYMK expression had short DSS than those of patients with low DTYMK expression in ACC (*p* = 0.007), KIRC (*p* = 0.001), KIRP (*p* = 0.004), LGG (*p* < 0.001), LIHC (*p* = 0.001), LUAD (*p* < 0.001), MESO (*p* = 0.002), PAAD (*p* = 0.007), SKCM (*p* = 0.002), and UVM (*p* = 0.001). The PFI analysis in Figure 7 revealed that DTYMK acted as a risk factor for patients with ACC (*p* < 0.001), KIRC (*p* = 0.004), LGG (*p* < 0.001), LIHC (*p* = 0.002), LUAD (*p* = 0.002), PAAD (*p* = 0.004), PRAD (*p* = 0.009), SKCM (*p* = 0.008), and UVM (*p* < 0.001). The results of Cox regression analyses were shown in Figure 8. The OS analysis indicated that DTYMK was a potential independent prognostic biomarker for patients with ACC, DLBC, LGG, LIHC, LUAD, MESO, SKCM, and UVM (Figure 8A). The DSS analysis suggested that DTYMK was a potential independent prognostic biomarker for patients with ACC, KIRP, LGG, LIHC, LUAD, MESO, SKCM, and UVM (Figure 8B). The PFI analysis revealed that DTYMK was a potential independent prognostic biomarker for patients with ACC, LGG, LIHC, LUAD, SKCM, and UVM (Figure 8C **)**. These data suggested that DTYMK expression was correlated with clinical prognosis in most tumors.

### 3.5 The correlation between deoxythymidylate kinase expression and clinicopathological characteristics

**FIGURE 9:**
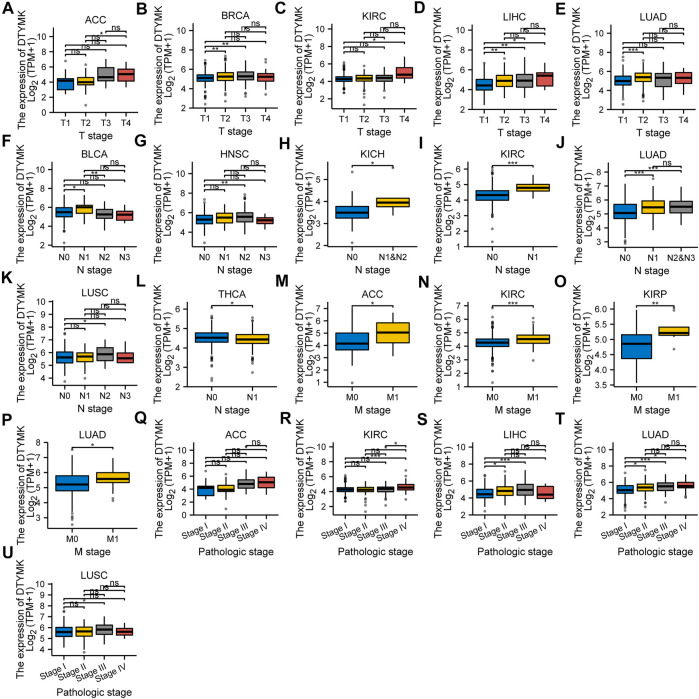
The correlation between DTYMK expression and clinicopathological characteristics. The correlation between DTYMK expression with the T stage in ACC, BRCA, KIRC, LIHC, and LUAD **(A–E)**, the N stage in BLCA, HNSC, KICH, KIRC, LUAD, LUSC, and THCA **(F–L)**, the M stage in ACC, KIRC, KIRP, and LUAD **(M–P)**, and TNM stage in ACC, KIRC, LIHC, LUAD, and LUSC **(Q–U)**. (ns, no significant; *, *p* < 0.05, **, *p* < 0.01, ***, *p* < 0.001)

We analyzed the mRNA expression of DTYMK and associated clinical data from a pan-cancer perspective from TCGA. The results from Figure 9 indicated that DTYMK expression was correlated with the T stage in ACC, BRCA, KIRC, LIHC, and LUAD (Figures 9A–E), with the N stage in BLCA, HNSC, KICH, KIRC, LUAD, LUSC, and THCA (Figures 9F–L), with M stage in ACC, KIRC, KIRP, and LUAD (Figures 9M–P), with TNM stage in ACC, KIRC, LIHC, LUAD, and LUSC (Figures 9Q–U). Furthermore, DTYMK expression was correlated with age in ESCA, KIRP, LGG, OV, SARC, STAD, and THYM (Supplementary Figures S3A–G), with gender only in HNSC and LUAD (Supplementary Figures S3H,I). To sum up, the results suggested that DTYMK might play a crucial role in the development of tumor progression.

### 3.6 PPI network and genetic alteration characteristics

**FIGURE 10:**
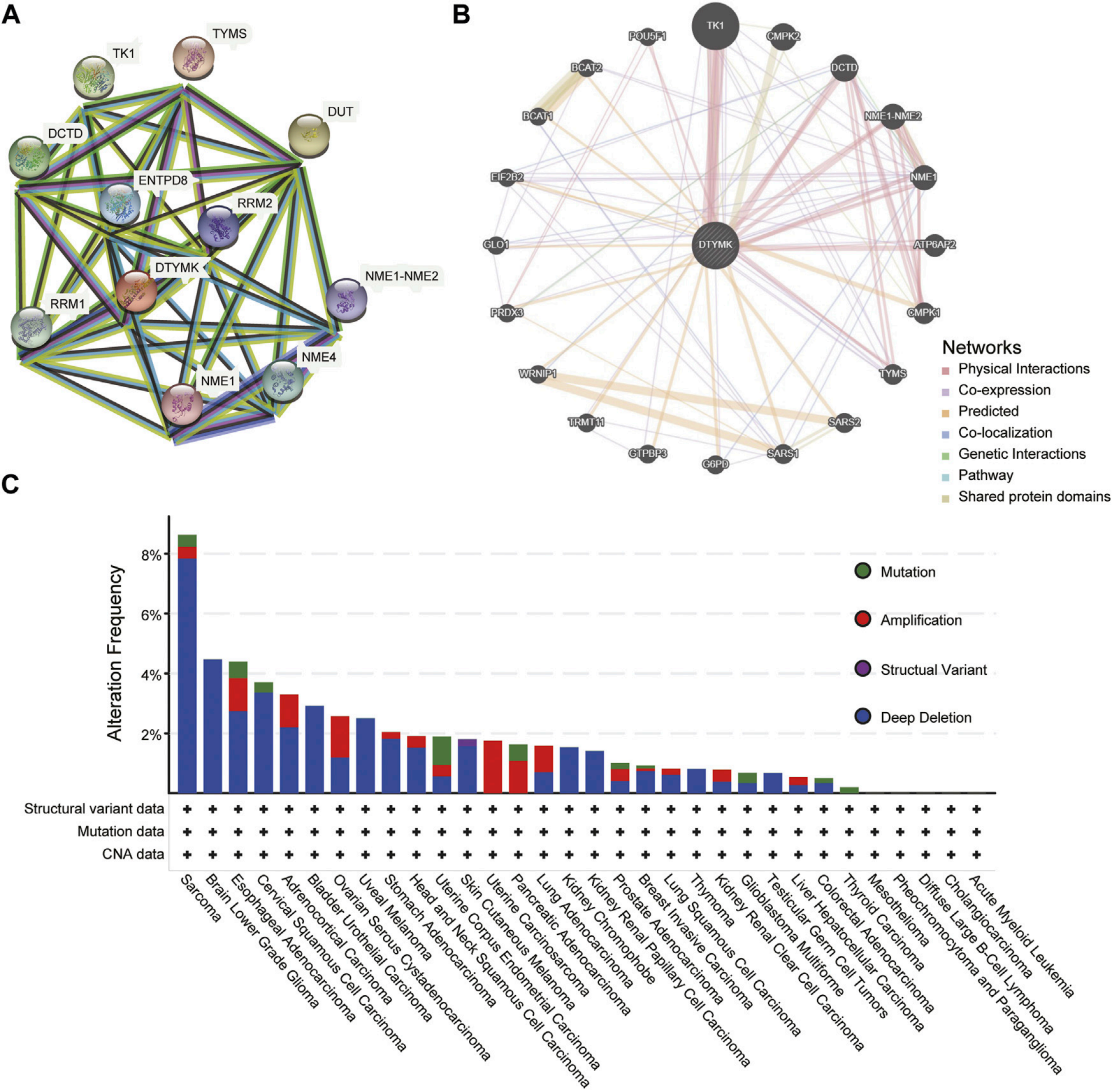
PPI network and genetic alteration characteristics. **(A)** A network of *DTYMK* and its co-expression genes. **(B)** GeneMANIA indicated *DTYMK* and its co-expression genes were involved in pyrimidine–containing compound biosynthetic process and pyrimidine nucleotide biosynthetic/metabolic process. **(C)** The genetic alteration characteristics of *DTYMK* across different tumors.

To predict a PPI network, we conducted an analysis on STRING and GeneMANIA. Figure 10A from STRING showed 10 co-expression genes of DTYMK and a PPI network. As shown in Figure 10B, results from the GeneMANIA suggested that DTYMK and its co-expression genes were involved in pyrimidine–containing compound biosynthetic process and pyrimidine nucleotide biosynthetic/metabolic process. Furthermore, we studied the genetic alteration characteristics of DTYMK across different tumors of the TCGA cohorts with the cBioPortal web. The result in Figure 10C showed that the most alteration frequency of DTYMK appeared in sarcoma patients with “deep deletion” as the main type. The “amplification” type was the primary type in OV, UCS, PAAD, and LUAD.

### 3.7 Expression profile of deoxythymidylate kinase in a single-cell level and its potential functional status in pan-cancer

**FIGURE 11:**
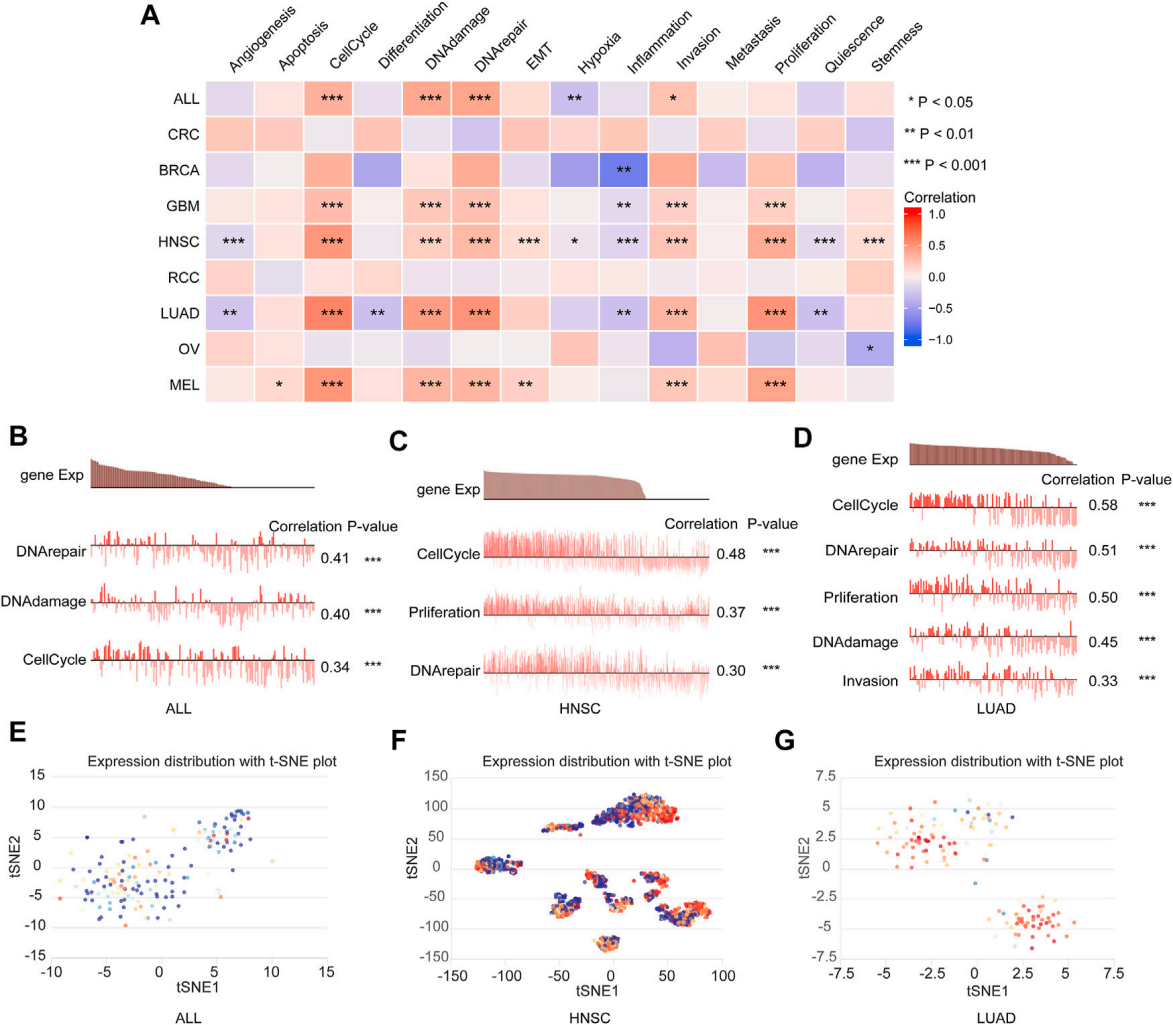
DTYMK expression and cancer functional states at a single-cell level. **(A)** DTYMK expression was correlated with cancer functional states in pan-cancer. **(B–D)** The association between DTYMK expression and cancer function in ALL, HNSC, and LUAD. **(E–G)** The t-SNE plot indicated DTYMK expression profile in single cells of ALL, HNSC, and LUAD. (ns, no significant; *, *p* < 0.05, **, *p* < 0.01, ***, *p* < 0.001).

To explore the expression profile of DTYMK at a single-cell level and its potential functional status in pan-cancer, we performed an analysis on CancerSEA. As shown in Figure 11A, the expression of DTYMK was significantly positively correlated with cell cycle, DNA damage, DNA repair and invasion in ALL, GBM, HNSC, LUAD, and MEL, DTYMK expression was positively correlated with EMT in HNSC and MEL, with proliferation in GBM, HNSC, LUAD, and MEL. Figures 11B,C indicated the association between DTYMK expression and DNA repair, DNA damage, and cell cycle in ALL and HNSC. Figure 11D indicated the association between DTYMK expression and cell cycle, DNA repair, proliferation, DNA damage, and invasion in LUAD. Furthermore, the expression distribution of DTYMK was shown in single cells of ALL, HNSC, and LUAD by a T-SNE plot (Figures 11E–G). Taking together, these results indicated that DTYMK might play an important role in tumor progression.

### 3.8 Relationship between deoxythymidylate kinase expression and immune infiltrates

**FIGURE 12:**
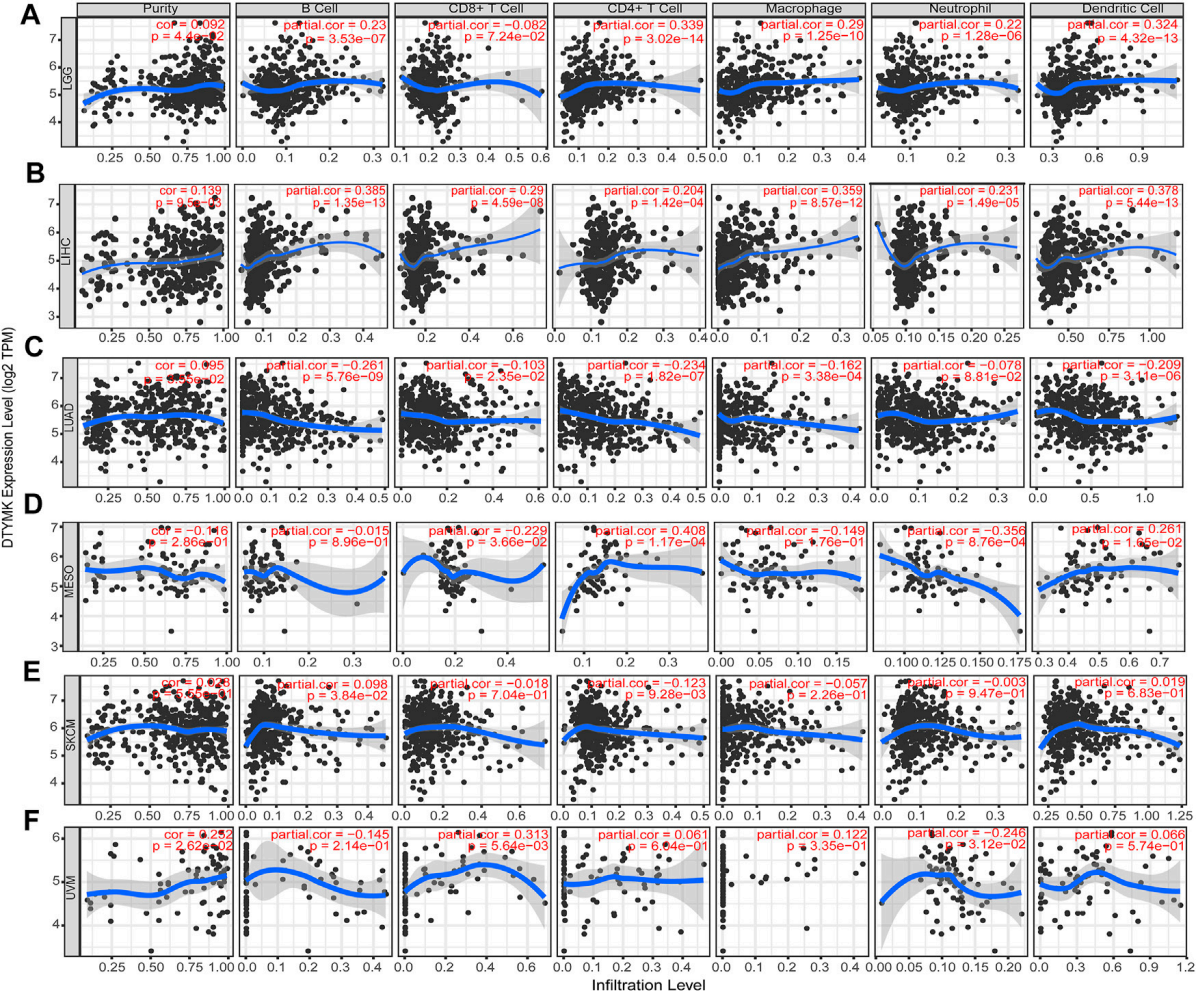
The relationship between DTYMK expression and immune infiltration levels. DTYMK expression was correlated with tumor-infiltrating immune cell levels in LGG **(A)**, LIHC **(B)**, LUAD **(C)**, MESO **(D)**, SKCM **(E)**, and UVM **(F)**.

**FIGURE 13:**
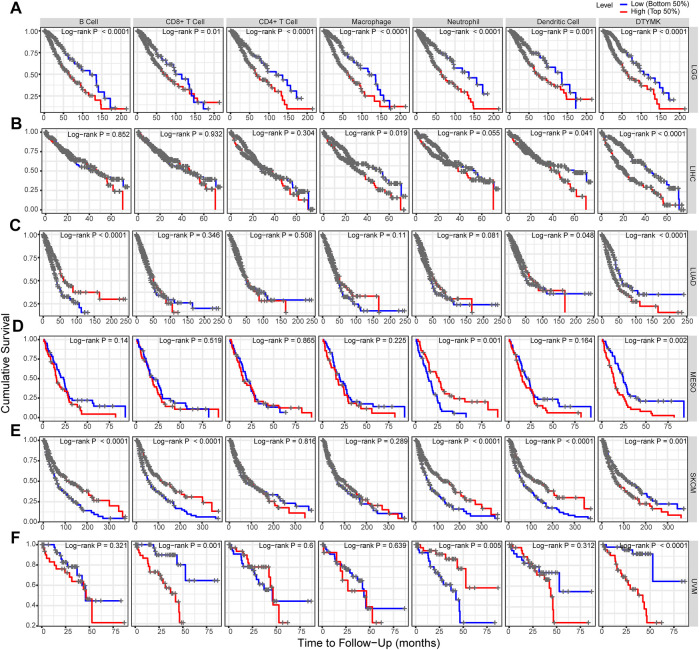
DTYMK regulates tumor-infiltrating immune cells level to affect prognosis. **(A)** High expression of B cells, CD8^+^ T cells, CD4^+^ T cells, macrophage, neutrophil, and dendritic cell was correlated with poor prognosis in LGG. **(B)** High expression of macrophage and dendritic cell was correlated with poor prognosis in LIHC. **(C)** Low expression of B cell and dendritic cells was correlated with poor prognosis in LUAD. **(D)** Low expression of neutrophil cells predicted poor prognosis in MESO. **(E)** Low expression of B cells, CD8^+^ T cells, neutrophil, and dendritic cells predicted poor prognosis in SKCM. **(F)** High expression of CD8^+^ T cells and neutrophil cells was correlated with poor prognosis in UVM.

Based on the expression of DTYMK was correlated with poor prognosis in LGG, LIHC, LUAD, MESO, SKCM, and UVM, we further assessed the relationship between DTYMK expression and immune infiltrates with TIMER in those tumors. As shown in Figure 12A, the “Gene” module suggested that DTYMK expression was positively correlated with infiltrating levels of B cells, CD4^+^ T cells, macrophage, neutrophil, and dendritic cells in LGG. DTYMK expression was positively correlated with tumor purity and infiltrating levels of B cells, CD8^+^ T cells, CD4^+^ T cells, macrophage, neutrophil, and dendritic cells in LIHC (Figure 12B). From the data in Figure 12C, DTYMK expression was negatively correlated with infiltrating levels of B cells, CD8^+^ T cells, CD4^+^ T cells, macrophages, and dendritic cells in LUAD. Figure 12D indicated that DTYMK expression was negatively correlated with tumor purity and infiltrating levels of CD8^+^ T cells, macrophages, and neutrophils, while positively correlated with infiltrating levels of CD4^+^ T cells and dendritic cells in MESO. As shown in Figure 12E, DTYMK expression was negatively correlated with infiltrating levels of CD4^+^ T cells in SKCM. In UVM, Figure 12F suggested that DTYMK expression was positively correlated with tumor purity and infiltrating levels of CD8^+^ T cells and macrophages, while negatively correlated with infiltrating levels of B cells and neutrophil cells in MESO. Moreover, to further confirm the impact of immune cell infiltration on the prognosis of those tumors, we drew Kaplan-Meier plots with the TIMER database. The “Survival” module analysis in Figure 13 indicated that patients with those tumors would share different prognoses according to the high/low expression of immune cell levels. Together these results showed that DTYMK had a potential role in regulating tumor-infiltrating immune cells level to further affect the prognosis of those tumors.

### 3.9 Correlation between deoxythymidylate kinase expression and gene markers of immune infiltrates

Finally, in order to further confirm the relationship between DTYMK expression and immune infiltrates, we used the TIMER database to explore the correlation between DTYMK expression and immunological markers in the above six tumors. We determined the correlation between the expression of DTYMK and immunological markers of immune infiltrates, including B cell, CD8^+^ T cell, T cell (general), macrophage (M1, M2), and dendritic cell. The correlation was adjusted by tumor purity. These results in Table 1 suggested that DTYMK expression was correlated with most immunological marker sets. In particular, DTYMK was significantly correlated with B cell markers (CD19, CD79A) in LGG, LIHC, LUAD, MESP, and SKCM, CD8^+^ T cell markers (CD8A, CD8B) in LIHC, SKCM, and UVM, T cell markers (CD3D, CD3E, CD2) in LGG, LIHC, SKCM, and UVM, Macrophage markers (NOS2, IRF5, PTGS2, CD163, VSIG4, MS4A4A) in LGG and UVM, Dendritic cell markers (HLA-DPB1, HLA-DPB1, HLA-DRA, HLA-DPA1, CD1C, NRP1, ITGAX) in LGG, LIHC, LUAD, and UVM. Our findings suggested that DTYMK expression was correlated with gene markers of immune infiltrates.

**TABLE 1:** Correlation analysis between DTYMK and immune infiltration markers in TIMER.

Description	Gene markers	LGG		LIHC		LUAD		MESO		SKCM		UVM	
		Cor	*P*	Cor	*P*	Cor	*P*	Cor	*P*	Cor	*P*	Cor	*P*
B cell	CD19	0.212	***	0.234	***	−0.117	*	−0.384	**	−0.126	*	0.023	8.41e-01
	CD79A	0.186	***	0.166	*	−0.125	*	−0.375	**	−0.165	**	−0.059	6.09e-01
CD8^+^ T cell	CD8A	−0.026	5.77e-01	0.226	***	0.04	3.77e-01	−0.096	3.85e-01	−0.123	*	0.507	***
	CD8B	−0.039	3.90e-01	0.286	***	0.106	1.62e-02	0.015	8.92e-01	−0.141	*	0.475	***
T cell (general)	CD3D	0.284	***	0.395	***	−0.03	5.06e-01	0.018	8.69e-01	−0.155	**	0.453	***
	CD3E	0.246	***	0.258	***	−0.163	**	−0.049	6.58e-01	−0.178	**	0.456	***
	CD2	0.272	***	0.281	***	−0.129	*	0.046	6.78e-01	−0.148	*	0.456	***
M1 Macrophage	INOS (NOS2)	−0.078	8.88e-02	−0.096	7.41e-02	0.001	9.85e-01	0.129	2.40e-01	0.065	1.63e-01	0.293	**
	IRF5	0.357	***	0.31	***	−0.019	6.73e-01	−0.107	3.28e-01	0.018	6.93e-01	0.586	***
	COX2 (PTGS2)	−0.184	***	0.034	5.28e01	0.01	8.28e-01	−0.312	*	0.069	1.40e-01	0.544	***
M2 Macrophage	CD163	0.156	**	0.064	2.33e-01	−0.105	2.00e-02	−0.037	7.35e-01	−0.057	2.27e-01	0.525	***
	VSIG4	0.173	**	0.146	*	−0.102	2.37e-02	0.042	7.01e-01	−0.06	2.01e-01	0.481	***
	MS4A4A	0.219	***	0.136	1.12e-02	−0.111	1.39e-02	0.011	9.23e-01	−0.122	*	0.541	***
Dendritic cell	HLA-DPB1	0.37	***	0.255	***	−0.389	***	−0.074	4.99e-01	−0.15	*	0.557	***
	HLA-DQB1	0.304	***	0.242	***	−0.312	***	−0.089	4.19e-01	−0.093	4.68e-02	0.512	***
	HLA-DRA	0.326	***	0.196	**	−0.32	***	−0.15	1.70e-01	−0.16	**	0.535	***
	HLA-DPA1	0.31	***	0.184	**	−0.378	***	−0.18	9.84e-02	−0.211	***	0.568	***
	BDCA-1 (CD1C)	0.123	*	0.134	1.31e-02	−0.43	***	−0.157	1.51e-01	−0.045	3.34e-01	0.166	1.49e-01
	BDCA-4 (NRP1)	0.153	**	0.087	1.09e-01	−0.163	**	0.153	1.62e-01	0.007	8.90e-01	0.569	***
	CD11c (ITGAX)	0.354	***	0.336	***	−0.139	*	−0.021	8.51e-01	−0.014	7.71e-01	0.658	***

Cor. correlation of Spearman’s R value; * *p* < 0.01; **p < 0.001; ***p < 0.0001, NA, no data.

## 4 Discussion

With the advancement in immunotherapy in recent years, the prognosis of cancer patients has been significantly improved (Hu et al., 2020). However, immune checkpoint inhibitors are not useful for all tumors and most tumor patients will develop resistance after the initial benefit (von Loga et al., 2020). The potential mechanisms underlying immunotherapy resistance are still poorly understood. It is reported that tumor-infiltrating immune cells were correlated with the prognosis of cancer patients and the antitumor efficacy of immunotherapy (Shi et al., 2020). A Previous study reported that the upregulation of DTYMK was correlated with unfavorable prognosis and the immune microenvironment in hepatocellular carcinoma (Guo et al., 2021). However, the role of DTYMK in tumor progression remains to be elucidated. In the present study, we systematically explored the expression, diagnostic and prognostic value, and correlation with immune infiltrates in a pan-cancer perspective.

In this study, we first explored the mRNA expression of DTYMK in 33 different cancer types using RNA-seq data from TCGA and integrated it with GTEx. TCGA data suggested that DTYMK is upregulated in 18 cancer types relative to normal tissues, including BLCA, BRCA, CESC, CHOL, COAD, ESCA, GBM, HNSC, KIRC, KIRP, LIHC, LUAD, LUSC, PRAD, READ, STAD, THCA, and UCEC. In comparison, DTYMK is only downregulated in KICH. Our findings agree with those studies that suggested DTYMK was increased in hepatocellular carcinoma (Guo et al., 2021; Sun et al., 2021), and non-small cell lung cancer (Liu et al., 2013). Based on DTYMK being abnormally expressed in a pan-cancer perspective, we conducted a ROC curve analysis to speculate the diagnostic value of DTYMK. The result suggested that the AUC value is more than 0.80 in BLCA, BRCA, CHOL, COAD, ESCA, HNSC, KIRP, LUAD, LUSC, READ, STAD, UCEC, and KICH, further suggesting DTYMK is a potential diagnostic biomarker to differentiate tumor tissues from normal tissues in these tumors.

Many studies reported that the upregulation of DTYMK was correlated with poor prognosis in various cancers. Zhou et al. (2021) reported that high expression of DTYMK significantly corresponded to the poor OS, DSS, and relapse-free survival (RFS) in hepatocellular carcinoma. Guo et al. (2021) suggested that increased DTYMK was correlated with poor OS and DFS in hepatocellular carcinoma. In the present study, our results indicated that DTYMK is a risk factor for poor OS in patients with ACC, KIRC, LGG, LIHC, LUAD, MESO, PAAD, SKCM, and UVM and a protective factor in patients with DLBC. For DSS, DTYMK expression is a risk factor for patients with ACC, KIRC, KIRP, LGG, LIHC, LUAD, MESO, PAAD, SKCM, and UVM. The PFI analysis suggested that DTYMK acted as a risk factor for patients with ACC, KIRC, LGG, LIHC, LUAD, PAAD, PRAD, SKCM, and UVM. These findings suggest that upregulation of DTYMK might act as a potential biomarker to identify tumor patients with poor clinical outcomes. Our finding that the mRNA expression of DTYMK is correlated with the T stage, N stage, M stage, and TNM stage in most tumors suggests DTYMK might play a crucial role in the development of tumor progression. To further investigate the detailed underlying mechanisms of oncogenic role in tumor progression, we analyzed the expression profile of DTYMK at a single-cell level and its potential functional status in pan-cancer. Our results showed that DTYMK expression is significantly positively correlated with cell cycle, DNA damage, DNA repair, and invasion. Our findings are consistent with that report by Zhou et al. that DTYMK can promote the cell cycle to enhance tumor growth and proliferation. Based on our data, we conclude that DTYMK might regulate the cell cycle to play a crucial role in the development of tumor progression and further lead to poor prognosis. However, this should be tested in other experiments.

In most solid tumors, the efficacy of immunotherapy is correlated with the tumor immune microenvironment, especially with infiltrating immune cells (Zhang et al., 2020). It is reported that the expression level of immune infiltration has been associated with prognosis in many cancer types (Guey et al., 2020), one underlying mechanism may be host immune defense by tumor-infiltrating immune cells against tumor progression (Sun et al., 2015). Moreover, immune-related genes can regulate systemic immune responses and are involved in the immune function of the body (Li et al., 2020). A previous study suggested that DTYMK might play an inhibiting effect on the immune microenvironment in the tumorigenesis of hepatocellular carcinoma (Guo et al., 2021). However, the correlation between DTYMK expression and tumor-infiltrating immune cells in pan-cancer has not been studied. In our study, we found that DTYMK expression is correlated with tumor-infiltrating immune cell levels by TIMER in LGG, LIHC, LUAD, MESO, SKCM, and UVM. Furthermore, a previous study suggested that the expression of tumor-infiltrating immune cells can predict the prognosis of LUAD patients (Lu et al., 2021). In this study, our data are consistent with this study and suggest that the six tumor-infiltrating immune cell expressions are correlated with the prognosis of patients with LGG, LIHC, LUAD, MESO, SKCM, and UVM. These findings lead us to speculate that DTYMK has a potential role in regulating tumor-infiltrating immune cells level to further affect the prognosis of these tumors, further suggesting that DTYMK is a potential therapeutic target for these tumors.

In conclusion, we applied a comprehensive pan-cancer analysis of DTYMK and found that DTYMK can be used as a potential diagnostic biomarker in most tumors. Our findings suggested that DTYMK expression is correlated with clinical prognosis, tumor progression and immune infiltrate. DTYMK has a potential role in regulating tumor-infiltrating immune cells level and might act as a potential target for immune therapy.

## Data Availability

The original contributions presented in the study are included in the article/Supplementary Material; further inquiries can be directed to the corresponding authors.

## Supplementary material

Supplementary Figure S1 Protein expression of DTYMK. The updownregulated protein of DTYMK was detected in BRCA **(A)**, and KIRC **(B)**. (ns, no significant; *, P < 0.05, ***, P < 0.001).

Supplementary Figure S2 Promoter methylation level of DTYMK. No significant difference of promoter methylation level were found in BRCA **(A)**, CHOL **(B)**, COAD **(C)**, CESC **(D)**, ESCA **(E)**, GBM **(F)**, KIRP **(G)**, LUSC **(H)**, PAAD **(I)**, PCPG **(J)**, READ **(K)**, SARC **(L)**, SKCM **(M)**, STAD **(N)**, THCA **(O)**, and THYM **(P).** (ns, no significant).

Supplementary Figure S3 The correlation between DTYMK expression and age and gender. **(A–G)** DTYMK expression was correlated with age in ESCA, KIRP, LGG, OV, SARC, STAD, and THYM. **(H,I)** DTYMK expression was correlated with gender only in HNSC and LUAD. (*, P < 0.05, **, P < 0.01, ***, P < 0.001).

Supplementary Table S2 The cut-off value, sensitivity, specificity, positive predictive value, negative predictive value, and Youden index of DTYMK in Pan-Cancer perspective.
