# Supplementary figures and images for: Pan-cancer analysis of prognostic and immunological role of DTYMK in human tumors
Source: Front Genet. 2022 Sep 8;13:989460. doi: 10.3389/fgene.2022.989460 (PMC9493117; doi:10.3389/fgene.2022.989460)

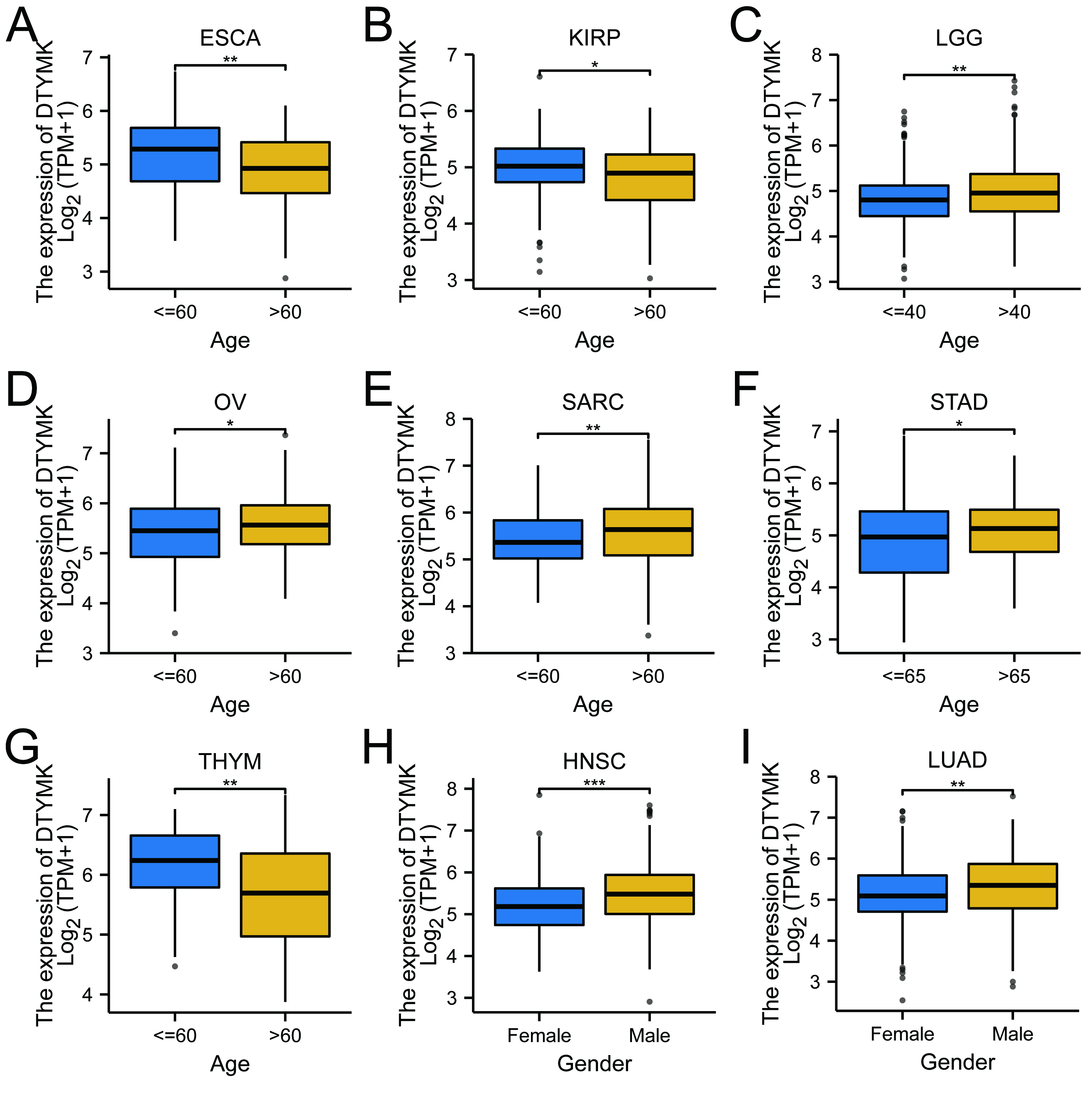

Supplement: Supplementary file 3 [file Image3.TIF]

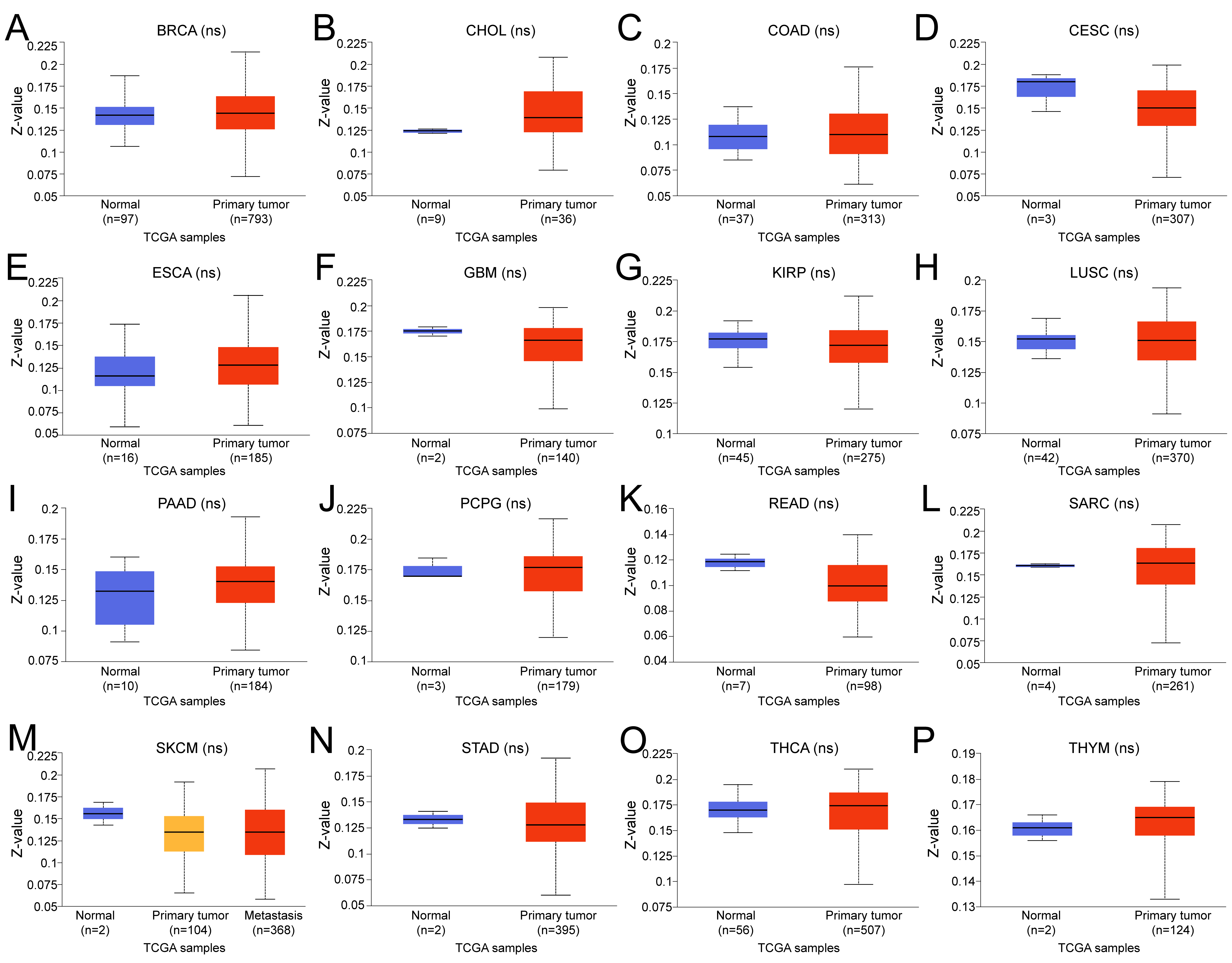

Supplement: Supplementary file 4 [file Image2.TIF]

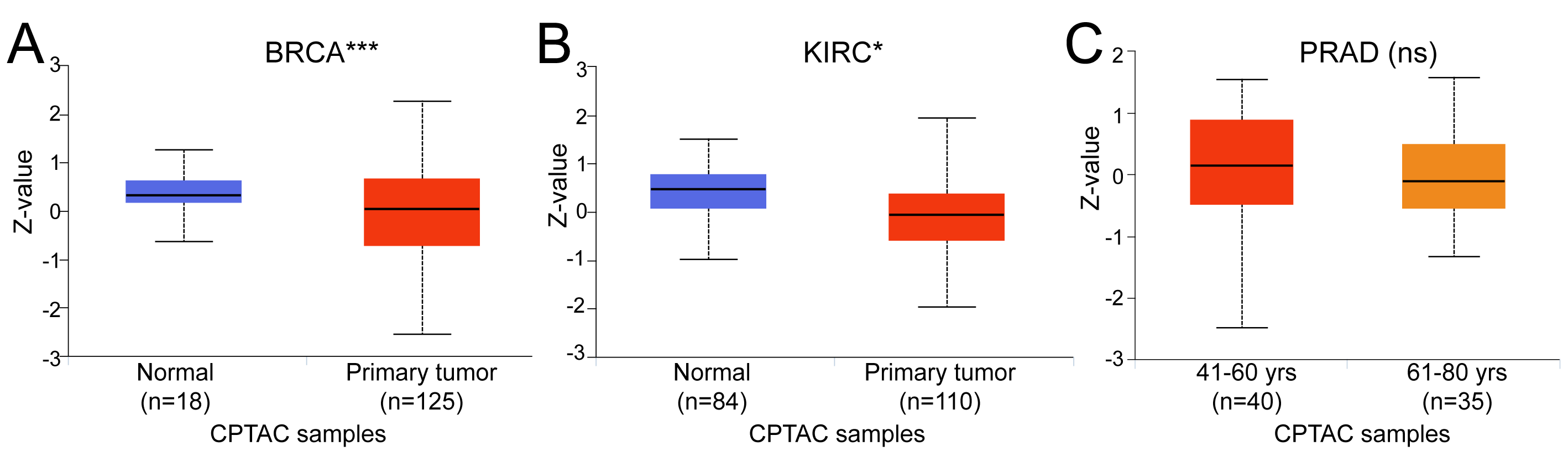

Supplement: Supplementary file 6 [file Image1.TIF]
