# Supplementary material for: Pan-cancer analysis of prognostic and immunological role of DTYMK in human tumors
Source: Front Genet. 2022 Sep 8;13:989460. doi: 10.3389/fgene.2022.989460 (PMC9493117; doi:10.3389/fgene.2022.989460)
Supplement: Supplementary file 5 [file Table7.DOCX]

| Tumor | cut-off | Sensitivity | Specificity | positive predictive value | negative predictive value | Youden index |
| --- | --- | --- | --- | --- | --- | --- |
| BLCA | 4.480 | 0.842 | 0.918 | 0.320 | 0.992 | 0.760 |
| BRCA | 4.587 | 0.823 | 0.859 | 0.373 | 0.979 | 0.682 |
| CHOL | 3.620 | 1.000 | 1.000 | 1.000 | 1.000 | 1.000 |
| COAD | 5.290 | 0.902 | 0.825 | 0.306 | 0.990 | 0.727 |
| ESCA | 4.110 | 0.818 | 0.932 | 0.450 | 0.987 | 0.750 |
| HNSC | 4.870 | 0.955 | 0.773 | 0.269 | 0.995 | 0.727 |
| KIRC | 3.967 | 0.542 | 0.790 | 0.257 | 0.928 | 0.332 |
| KIRP | 4.778 | 0.938 | 0.651 | 0.229 | 0.989 | 0.558 |
| LIHC | 3.554 | 0.940 | 0.922 | 0.618 | 0.991 | 0.862 |
| LUAD | 4.633 | 0.949 | 0.806 | 0.350 | 0.993 | 0.755 |
| LUSC | 4.772 | 0.959 | 0.934 | 0.588 | 0.996 | 0.893 |
| PRAD | 4.728 | 0.692 | 0.733 | 0.213 | 0.958 | 0.426 |
| READ | 5.028 | 0.800 | 0.928 | 0.400 | 0.987 | 0.728 |
| STAD | 3.917 | 0.781 | 0.936 | 0.510 | 0.980 | 0.717 |
| THCA | 4.466 | 0.897 | 0.522 | 0.176 | 0.978 | 0.418 |
| UCEC | 4.981 | 0.914 | 0.842 | 0.269 | 0.994 | 0.757 |
| KICH | 3.986 | 1.000 | 0.862 | 0.727 | 1.000 | 0.862 |
