# Supplementary material for: Pan-cancer analysis of prognostic and immunological role of DTYMK in human tumors
Source: Front Genet. 2022 Sep 8;13:989460. doi: 10.3389/fgene.2022.989460 (PMC9493117; doi:10.3389/fgene.2022.989460)
Supplement: Supplementary file 9 [file Table6.DOCX]

**Supplementary Table 1** Abbreviations of tumor names.

| **Abbreviations** | **Tumor** |
| --- | --- |
| ACC | Adrenocortical carcinoma |
| BLCA | Bladder Urothelial Carcinoma |
| BRCA | Breast Invasive Carcinoma |
| CESC | Cervical Squamous Cell Carcinoma and Endocervical Adenocarcinoma |
| CHOL | Cholangiocarcinoma |
| COAD | Colon Adenocarcinoma |
| DLBC | Lymphoid Neoplasm Diffuse Large B-cell Lymphoma |
| ESCA | Esophageal Carcinoma |
| GBM | Glioblastoma mutiforme |
| HNSC | Head and Neck Squamous Cell Carcinoma |
| KICH | Kidney Chromophobe |
| KIRC | Kidney Renal Clear Cell Carcinoma |
| KIRP | Kidney Renal Papillary Cell Carcinoma |
| LAML | Acute Myeloid Leukemia |
| LGG | Brain Lower Grade Glioma |
| LIHC | Liver Hepatocellular Carcinoma |
| LUAD | Lung Adenocarcinoma |
| LUSC | Lung Squamous Cell Carcinoma |
| MESO | Mesothelioma |
| OV | Ovarian Serous Cystadenocarcinoma |
| PAAD | Pancreatic Adenocarcinoma |
| PCPG | Pheochromocytoma and paraganglioma |
| PRAD | Prostate Adenocarcinoma |
| READ | Rectum Adenocarcinoma |
| SARC | Sarcoma |
| SKCM | Skin Cutaneous Melanoma |
| STAD | Stomach Adenocarcinoma |
| TGCT | Testicular Germ Cell Tumors |
| THCA | Thyroid carcinoma |
| THYM | Thymoma |
| UCEC | Uterine Corpus Endometrial Carcinoma |
| UCS | Uterine Carcinosarcoma |
| UVM | Uverl melanoma |
